# Combination of Material Processing and Characterization Methods for Miniaturization of Field-Effect Gas Sensor

**DOI:** 10.3390/s23010514

**Published:** 2023-01-03

**Authors:** Nikolay Samotaev, Artur Litvinov, Konstantin Oblov, Maya Etrekova, Boris Podlepetsky, Pavel Dzhumaev

**Affiliations:** Micro- and Nanoelectronics Department, National Research Nuclear University MEPhI (Moscow Engineering Physics Institute), Kashirskoe Highway 31, 115409 Moscow, Russia

**Keywords:** field-effect gas sensor, laser micromilling, pulse laser deposition, metal–ceramics package, tomography

## Abstract

The technological approach for the low-scale production of field-effect gas sensors as electronic components for use in non-lab ambient environments is described. In this work, in addition to the mechanical protection of a gas-sensitive structure, an emphasis was also placed on the very topical issue of thermal stabilization around the one temperature point, even if it is several degrees higher than the surrounding one, which will probably also be useful for any type of application for many types of field-effect sensors. Considerable attention was paid to the characterization of the results obtained by various invasive and non-invasive methods for diagnosing the manufactured construction. The technology described in this article occupies an intermediate position between laboratory samples tested in clean rooms with stable ambient atmospheres, and experimental and small-scale production sensors designed for real operating conditions to solve the narrow application of measuring low concentrations of hydrogen.

## 1. Introduction

As a rule, the miniaturization of elements in microelectronics is directly related to the conditions of their functioning in the ambient environment. The simplest case is digital and analog microcircuits, the operation of which requires no access the external environment. There are many technologies and versions of packages for placing a microchip in them and then sealing the package from the effects of the external environment [1]. Usually, for small batches of such components, there are a wide range of metal–ceramic packages for sale from well-known manufacturers [2]. The next level of problems occurs for solid-state sensor elements, where they need to interact with an external environment; this is either optical radiation or the direct mass transfer (gas or liquid) of the ambient environmental inside the package. Optical sensor elements usually use modifications of standard metal–glass [3] or ceramic–metal [4] packages with sealed optical windows made from various transparent materials. A segment remains that is difficult from the point of view of the mass production market of solid-state sensors that measure different concentrations of substances in aggressive environments. Generally, such sensors are used in industrial applications and their number is not high. A series of sensors can consist of several hundreds or thousands of copies, but the requirements for the geometry of the package and the resistance of its materials to external factors differ from anything existing on the microelectronics mass market. An example of such a solid-state sensor is the field-effect gas sensor described in our work [5] for measuring hydrogen dissolved in transformer oil. The market for such sensors is not large (for Russia it is several thousand per year), but the miniaturization of sensors is necessary because the small package dimensions allow the sensors to be used as submersible elements in oil tanks against convenient flow systems [6].

A possible lead example for the miniaturization of field-effect gas sensor packages was used in [7,8,9], where the metal–glass package [9] was replaced with a metal–ceramic package [7] made using LTCC (low-temperature co-fired ceramics) technology [8]. Working with multilayer green tape ceramics implies the availability of equipment [10] and the availability of a compatible set of materials (ceramics tape and metallization and glass insulator pastes), which are produced by a limited number of companies in the world [11]. This makes LTCC technology complicated [12], expensive, and slow (the shelf life of raw non-sintered materials is several months, and it is required to wait for the delivery of new material). On the contrary, if the ceramic material is already sintered, then its storage time is not limited, and the availability is much wider than that of green tape ceramics. Motivated by the above arguments, as an alternative to LTCC technology, in our works [13,14,15,16] we promote the technology of monolithic sintered ceramics laser processing to obtain metal–ceramic cases of a small series without incurring large resource and time costs. In this article, the step-by step strategy and results for the miniaturization of capacitive field-effect gas sensors with the transition from a form factor based on a bulky glass–metal TO-8 Ø12.7 × 7.5 mm package to a smaller planar custom 5.2 × 6.4 × 2.0 mm metal–ceramic package manufactured using adaptive laser micromilling technology is described.

**Figure 1:**
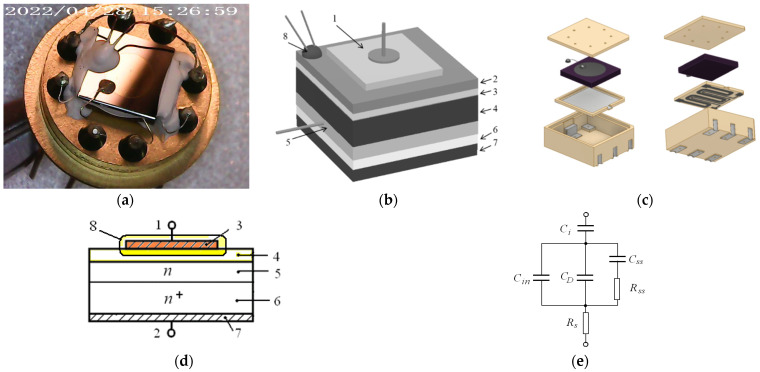
Field-effect gas sensor. (**a**) Image of the field-effect gas sensor based on a Si chip, assembled in the TO-8 package. (**b**) Schematic of the field-effect gas sensor based on a Si chip, assembled in the TO-8 package, including a capacitor, the substrate of which is an n-type silicon wafer “4” that is coated on one side with a SiO_2_ “3” film and an additional Ta_2_O_5_ “2” layer deposited on the dielectric by the laser deposition catalytic metal Pd “1”, to which an external metal electrode is glued. The second electrode is glued to the metal layer “5” deposited on the reverse side of the Si wafer. The gas-sensitive structure with a temperature-measuring thermistor “8” is located on an glass insulating plate “6” separating it from the film resistive heater “7”. (**c**) A 3D model of the metal–ceramic package layout: beige—ceramics from Al_2_O_3_, light and dark gray—silver and platinum metallization, violet—Si crystal. (**d**) The simplest structure of a field-effect capacitor gas sensor based on an n-type semiconductor: “1“—working electrode lead of “3”, “2”—lead of Ohmic contact “7”, “4”—thin dielectric film (50–200 nm thick), “5”—high-resistance Si region (donor concentration: N_D_ < 10^16^ cm^−3^), “6”—low-resistance semiconductor region (concentration: N_D_ > 10^18^ cm^−3^), “8”—gas-sensitive region. (**e**) Simplified equivalent electrical circuit field-effect gas sensor based on a Si chip.

If the technological prerequisites that formed the basis of this work are described above, then below is the physical factor on which the stability of the developed sensors was based. The first important aspect for the correct physical functioning of the sensor is its operating temperature; this can be demonstrated through the equivalent electrical circuit, as shown in Figure 1e. The working temperature is the only non-technological factor that affects the sensor response. The main informative characteristic of gas sensors based on the field effect is its capacitance–voltage characteristic (CVC), which is the dependence of capacitance (C) on voltage (V). The voltage (V) and the capacitance (C) can be represented in the general form as functions of the surface potential (φ_s_), respectively, by the formulas

*V* (φ*_s_*) *=* φ*_ms_* − [*Q_te_* + *Q_ss_* (φ*_s_*) + *Q_s_* (φ_s_)]/*C_i_ +* φ*_s_*, (1)

*C* (φ*_s_*) = *C_i_* [*C_in_* (φ*_s_*) + *C_D_* (φ*_s_*) + *C_ss_*(φ*_s_*)]/[*C_i_* + *C_in_* (φ*_s_*) + *C_D_*(φ*_s_*) + *C_ss_*(φ*_s_*)]*,* (2)

where φ*_ms_* is the potential of the work function difference between the metal and the semiconductor (*q*φ*_ms_*); the values of *Q_te_*, *Q_ss_,* and *Q_s_* are the effective charge densities in the dielectric, in the dielectric–semiconductor interface, and in the semiconductor; and the values of *C_i_*, *C_in_, C_D_,* and *C_ss_* are the capacitances of the dielectric film, the inversion layer, the space layer region of the semiconductor, and the effective capacitance of the surface states at the dielectric–semiconductor interface. The CVC of a field-effect gas sensor is shifted towards negative voltages parallel to the initial CVC by Δ*V* (*N*) under the action of a gas concentration (*N)*. The voltage (*V)* can be presented as *V* = *V*_0_ − Δ*V*(*N*). The dependence Δ*V*(*N*) = Δφ*_ms_*(*N*) − Δ*Q_te_*(*N*)/*C_i_* determines the intrinsic field-effect capacitor gas sensitivity (*S_V_* = d(Δ*V*)/d*N)* at temperatures from 25 °C to 180 °C. For field-effect capacitors with the Pd-Ta_2_O_5_-SiO_2_-Si structure presented in Figure 1a, within the limits of hydrogen concentrations from units of ppm to 1 vol % and at the temperatures from 25 °C to 180 °C, the experimental dependences Δ*V* (*N*) and *S_V_* (*N*) can be approximated as

Δ*V* (*N*) = Δ*V_m_* · [1 − exp(− *k_N_* ·*N*)] и *S_V_* = *k_N_* · Δ*V_m_* · exp(− *k_N_* ·*N*), (3)

where the parameters Δ*V_m_* and *k_N_* depend on the operating temperature (*T)*, the production technology, and the thicknesses of the Pd and Ta_2_O_5_-SiO_2_ films. The voltage *V*_0_, being equal to the initial value of *V* (for *N* = 0), is presented as

*V*_0_ = |φ*_s_*| + *a*·{|φ*_s_*| + φ*_T_* ·exp[(|φ*_s_*| − 2φ*_s_*_0_)/φ*_T_*)]}^1/2^ + φ*_ms_*_0_ − (*Q_te_*_0_ + *Q_ss_*_0_)/*C_i_*, (4)

where *a* = [2ε*_s_*·*qN_D_ d* ^2^/(ε_0_ε^2^)] ^1/2^, φ*_s_*_0_ = φ***_T_*** ln(*N_D_/n_i_*) is the donor-level potential, the potential φ***_T_*** = *kT/q*, *k* is the Boltzmann constant, *T* is the absolute temperature, and q is the electron charge. To avoid the influence of capacitances *C_ss_* and *C_in_* in capacity field-effect sensors, we selected the working area for high-frequency CVC characteristics (*f* > 50 kHz) in modes of depletion and weak inversion. In this domain, the dependence *C*(*V*) is defined as

*C*(*V*) = *C_i_* ·*C_D_*(*V*)/[*C_i_* + *C_D_*(*V*)] = *C_i_*/[1 + *F_C_* (*V*)], (5)

*F_C_* (φ*_s_*(*V*)) = 2·{|φ*_s_*| + φ*_T_* ·exp[(|φ*_s_*| − 2φ*_s_*_0_)/φ*_T_*)]}^1/2^/{*a* + *a* ·exp[(|φ*_s_*| − 2φ*_s_*_0_)/φ*_T_*)]}*,* (6)

|φ*_s_*| = |*V*| + 0.5*a*^2^ − 0.5*a* (*a*^2^ + 4|*V*|) ^1/2^*,* (7)

The presented formulas can be the basis for assessing the effect of chip temperature and its fluctuations on the gas sensitivity, errors, and stability of field-effect sensors. In each of the above factors, the stabilization of the operating temperature is important, and therefore the role of the package with a built-in heater is paramount.

## 2. Materials and Methods

### 2.1. The 3D Modeling and Digital Flow

While [13] demonstrated the practical possibility of manufacturing a 9.0 × 9.0 × 1.0 mm metal–ceramic package of a simple design without vias, in the current work a more miniature design of 5.2 × 6.4 × 2.0 mm with vias and final metallization was chosen. Figure 1a presents an image of the initial sensor in TO-8 used in [5] for hydrogen concentration control in oil-filled power transformers; the 3D model of the designed metal–ceramic package is shown in Figure 1c. The design feature of the metal–ceramic package is the high degree of 3D integration of the sensor components. A heater (combined with a resistive thermometer) and a gas-sensing chip based on a field-effect semiconductor structure are presented in Figure 1b. The complexity of the manufactured metal–ceramic package structure required subsequent characterization by tomography methods, the results of which are discussed in the next section.

**Figure 2:**
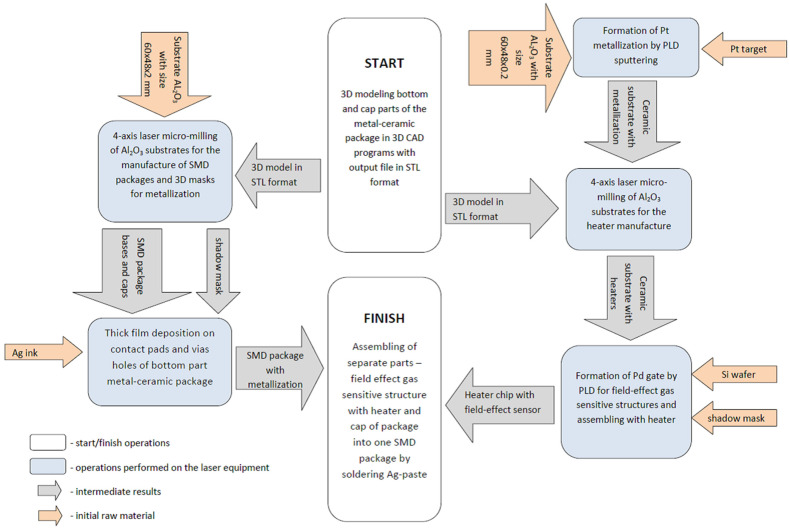
Chart of the technological process steps for the rapid prototyping of a field-effect gas sensor in a metal–ceramic package.

The ceramic material chosen for the package and heater was 96% alumina, which is often used in electronics. The main bottom part of the package was fabricated on a substrate with a size of 48.0 × 60.0 × 2.0 mm, and the heater was fabricated on a substrate with a size of 48.0 × 60.0 × 0.25 mm. The metallization of the heater and the thermistor measuring the working temperature was performed using vacuum pulse laser deposition with a pure platinum target. The internal and external metallization of the package was carried out with a silver ink for ink-jet printing because the low viscosity of ink gives perfect penetration in via holes compared with convenient post-firing silver-based pastes. A typical scheme of technology flow for miniature field-effect gas sensors has the steps presented in Figure 2.

To manufacture various parts of the developed 3D sensor model, a fiber laser with a power of 20 W, an adjustable pulse duration in the range of 50–200 ns, and a wavelength of 1.064 μm, controlled by specially developed software [16], was used. This approach allowed us to combine micromachining processing with the online measurement of the geometric parameters of the manufactured device with its 3D model and be confident in the quality of the result. At the stage of 3D modeling, it was necessary to add jumpers to the model, which held it in the array of substrates (framework). The point where the jumper touched the 3D model depended on the size of the model. For our models, shown in Figure 1c, there was a pyramid with a 200 × 200 µm square apex (where it attached to the chip). The preparation time for milling a 3D object of this size using specialized software was less than a minute. The process of the laser milling of Al_2_O_3_ ceramics was carried out at a rate of ~50 mm^3^/h. Depending on the required surface quality, the milling speed could be increased or decreased. Once started, the process could be paused at any time to view the machined object with a microscope at 400–2000× magnification or measure the roughness/height of the machined layer using the point laser profilometer built into the adaptive laser machine and then continue milling from the stop point.

Moreover, an additional positive moment accelerating the process of designing and manufacturing field-effect sensors was the use of the same laser processing complex for the manufacture of 2D metal masks for the laser deposition of gas-sensitive structures on silicon substrates and 3D masks for the ink-jet coating of silver metallization. The importance of the masks for silver ink-jet coating plating lied in the possibility of the automated deposition of metallization on the ends of the ceramic package, which in hand-made mode takes a lot of hard work and time.

### 2.2. Field-Effect Gas-Sensing Structure

The gas-sensitive structures were fabricated by the pulsed laser deposition technology, which involved the evaporation of the palladium target material with a focused laser beam and the subsequent deposition of the ejected material onto a substrate through the shadow mask. The steel shadow mask was fabricated using the adaptive laser micromilling technology, which was also applied for ceramic micro-milling. An aluminum–yttrium garnet laser (λ 1.06 µm) was used. The laser was operated in the Q-switched mode with a pulse duration of 10 ns, pulse energy of 0.1 J, and pulse frequency of 25 Hz. The Pd gate thickness was about 150–200 nm. The deposited metal film could spontaneously change its microstructure, especially when the sensor was operated at elevated temperatures. However, the finely dispersed film structure could be stabilized by annealing at high temperatures. Therefore, sensors that were ready and had their quality verified were annealed in air at T ≥ 150 °C for 100 h. The annealing temperature was set to the operating temperature of the sensors in the gas analyzers. The annealing time was enough for the stabilization processes to complete in principle.

Due to the semiconductor, the capacitive field-effect sensor had a variable electrical capacitance, which depended on the voltage applied to the plates, the temperature, and the chemical composition of the analyzed gas sample. The sensor’s operating temperature was maintained using a miniature heater (usually in the range from 100 °C to 150 °C) and stabilized with an electronic unit with a thermistor. The electronic unit also provided a stable voltage value on the capacitor, thereby setting the operating point on the sensor capacitance–voltage characteristic (the so-called dependence of the sensor capacitance on the bias voltage). Thus, when gas molecules were adsorbed on the gate surface, the charges in the semiconductor were redistributed and the capacitance–voltage characteristic curve shifted along the voltage axis. This led to a change in the sensor’s electrical capacitance proportional to the gas concentration, which was the principle of the sensor operation.

### 2.3. Package Assembling

**Figure 3:**
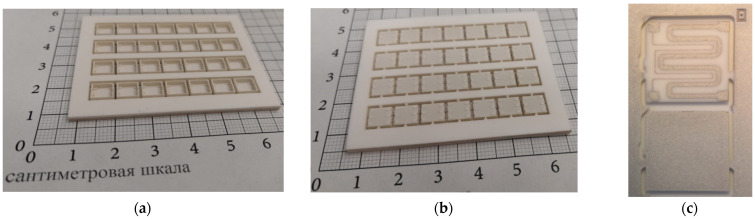
Image of bottom parts of packages and platinum heater fabricated in a 48.0 × 60.0 × 2.0 mm substrate with laser micromilling: (**a**) substrate with raw 7 × 4 bottom part of package—top view; (**b**) substrate with raw of 7 × 4 bottom part of package—backside view; (**c**) heater view before and after metallization laser ablation according to a 3D model of heater and thermistor metallization topology.

Assembling a sensor into the metal–ceramic package is a responsible operation. You can ideally carry out all of the above technological steps for manufacturing each part of the package but cannot achieve synergistic effects from all of them. Figure 3 shows the substrate on which metal–ceramic packages were assembled. Moreover Figure 3 presents images of a heater before and after metallization laser ablation according to a 2D model of microheater and thermistor metallization topology. The assembly of an array of packages on the frame has the advantage that there is no need to manipulate small objects, and it simplifies the manufacture of various assistant tools for assembly operation.

**Figure 4:**
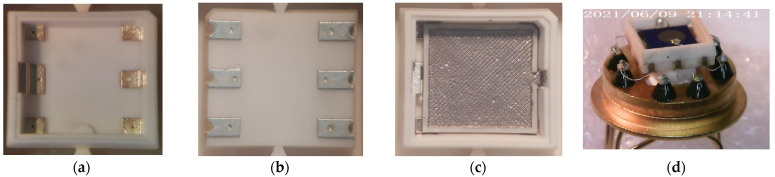
Assembling the sensor into the miniature metal–ceramic package: (**a**) bottom part of package with silver metallization—top view; (**b**) bottom part of package with silver metallization—top view; (**c**) assembling heater with bottom part of package by soldering; (**d**) final image of the field-effect gas sensor based on a Si chip assembled in the metal–ceramic package glued into a TO-8 package holder for tomography and gas-sensitive tests.

The sequence of assembling the sensor into a ceramic–metal package is shown in Figure 4. The assembly was carried out sequentially according to the 3D model shown in Figure 1. Ensuring the electrical contact of the sensor parts relative to each other during assembly was carried out by silver soldering. In the end, the field-effect gas sensor based on a Si chip, assembled in the metal–ceramic package glued into a TO-8 package holder for future characterization, was complete and fully functional. The main criterion for examining the quality of the assembly was the correctness of the soldering and the filling of the vias with metal—critical areas for the flow of electric current.

## 3. Results

The characterization of the results of this work included two main areas: online optical research methods that helped in the manufacture of the case (optical and laser profilometry integrated into the production unit) and off-line research methods confirming the effectiveness of the online methods and the full working functionality of the sensor in a manufactured metal–ceramic package (SEM, tomography, and thermal imaging). We further describe the methods that helped us to confirm the full functionality of the product, and we omit the geometric parameters controlled by the widely available methods, as they were already described in [16].

### 3.1. Thermal Characterization

The feature of the manufactured sensor should be the temperature distribution generated by the heater along the perimeter of the package. The thermal characterization used an FLIR T650SC IR camera and the measurement of power consumption vs. temperature with direct measurements using a miniature Pt-1000 sensor [17] glued to the MIS structure.

**Figure 5:**
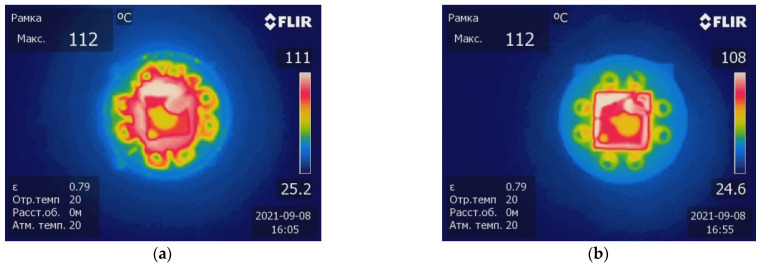
The results of the thermal imaging of MIS sensors with different heater configurations at an operating temperature of 100 °C: (**a**) standard assembly in a glass–metal package; (**b**) new assembly in a planar SMD package.

It was also shown in [13] that, compared with a bulky glass–metal package, it was possible to halve the power consumption of the MIS sensor and reduce its thermal inertia. A comparison of the results of the thermal imaging for the old and new sensor package designs is shown in Figure 5.

### 3.2. Laser Characterization

**Figure 6:**
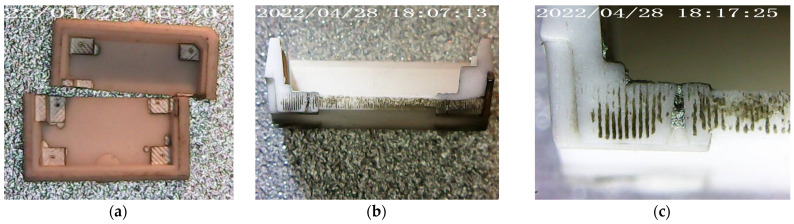
The results of laser cutting the bottom part of a metal–ceramic package on the via hole line: (**a**) the bottom part of a metal–ceramic package, cut into two parts by a laser; (**b**) cross-section of the bottom part of a metal–ceramic package; (**c**) the enlarged photo of a via not completely filled with silver ink.

Optical profilometry methods allow you to have on-line control of the geometric dimensions of ceramic blanks, but they do not penetrate the ceramic blanks, especially via holes or assembled blanks by soldering parts of packages. The use of a laser micromilling technique makes it possible to quickly control the dimensions and effectiveness of technological operations hidden from optics (for example, filling a hole with metal). With a 3D model in hand, you can very precisely assign lines along which the manufactured package will be cut for visual quality control under a microscope. As an example, we can cite Figure 6, which demonstrates the study of filling the transitional hole of a package with silver ink by cutting it with a laser on two symmetrical parts.

Of course, laser cutting gives an instant result in terms of the characterization of the metal–ceramic package, but it has a negative effect: laser irradiation destroys the package, and under certain conditions the destruction of the structure can still distort the result of characterization. For example, if fusible materials are used, they can evaporate from the via holes due to indirect heating due to the propagation of thermal waves through the ceramic material or remaining traces of contamination after laser cutting, as shown in Figure 6c. Therefore, it is important to always compare the methods of destructive and non-destructive testing in parallel with each other. For example, to combine the laser cutting of package parts and industrial tomography, the results of the study of the manufactured metal–ceramic package are discussed further.

### 3.3. Tomography Characterization

**Figure 7:**
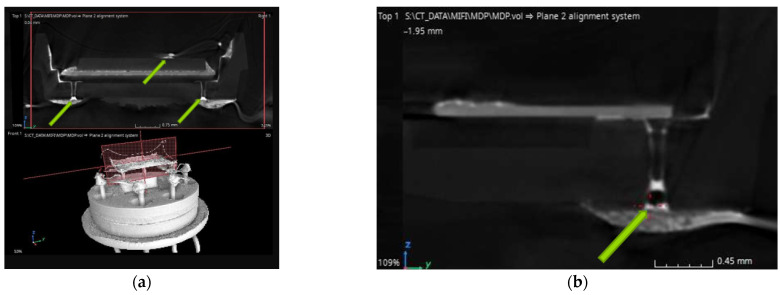
The reverse design of a 3D digital model of a metal–ceramic sensor package mounted on a TO-8 package holder: (**a**) arrows indicate soldering points; (**b**) the sections show the via holes in the ceramic base and the partial filling of the via holes by solder.

For the final characterization, a WG v|tome|x m300 industrial tomograph research system with a voxel resolution of µm 7.3 was used. The creation of a full-size tomographic 3D model for the TO-8 [18] package form-factor size took 1.5 h. The reverse design of a 3D digital model metal ceramic sensor package mounted on a TO-8 holder is presented in Figure 7.

The tomography model clearly shows the differences between materials with different atomic masses, which illustrates the various materials included in the metal–ceramic case, and, on the contrary, an excessive amount of metal in the old type of glass–metal package prevented the formation of a high-resolution model. The contrast in the materials and the high resolution of tomography allowed us to check the fill levels of via holes when soldering the metal–ceramic sensor package to the printed circuit board. Figure 6b indicates possible errors in the design of the metal–ceramic package in the form of the remaining open via holes capable of absorbing liquid solder due to the capillary effect. Moreover, such studies make it possible to reveal hidden places of the rupture of metallization due to the effects of thermo-electromigration [19] occurring in silver materials at high temperatures and high currents, characteristic of a wide class of microheaters for using solid-state gas sensors [20]. It must be taken into account that the tomography method also has its drawbacks; long exposure in highly focused X-rays negatively affects the properties of materials: firstly, a semiconductor, as a gas-sensitive element (charging dielectric layer), and secondly, ceramics, the glass in the composition, which visually darkens.

## 4. Discussion

**Figure 8:**
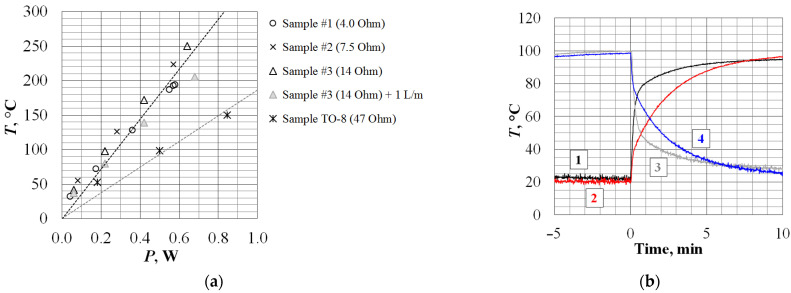
Comparative tests of the energy consumption and thermal inertia of the new (SMD) and old (TO-8) designs of gas sensors. (**a**) Plot with the power consumption of gas sensors required to maintain the operating temperature, including when blowing the sensor with an air flow of 1 l/min. (**b**) Plot of the time required to heat MIS sensors to 100 °C at a constant heater supply voltage (without thermal control) (1—SMD and 2—TO-8) as well as the duration of cooling to room temperature in the absence of voltage on the heater (3—SMD and 4—TO-8).

After the manufacture of the complete prototype of the field-effect gas sensor in the SMD package, energy consumption and thermal-inertial tests were carried out. We tested three samples of heaters with different values of resistance: 4, 7.5, and 14 ohms (the difference in the thickness of the deposited platinum was the difference in the duration of the PLD process). The following dependence of the heating temperature on the power of a ceramic-based platinum heater was established in comparison with the initial design of a 47-Ohm-thick film resistive heater, as shown in Figure 8a [5,21]. Preliminary tests showed that the power consumption required to heat the sensor operating temperature to 150 °C was halved. At the same time, the heating time to the operating temperature was reduced and remained smoother, and the cooling time was also halved (graph in Figure 8b).

**Figure 9:**
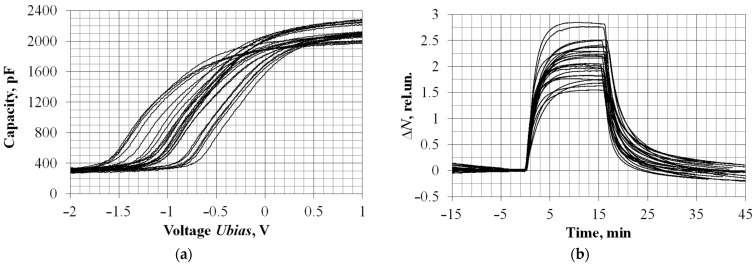
The test results of the operating parameters for assembled miniature capacitive sensors. (**a**) Capacitance–voltage characteristics at an operating temperature of 100 °C, measured on an AMM-3068 RLC meter with a test signal frequency of 20 kHz and an amplitude of 50 mV. (**b**) Dynamics of changes in sensor responses when a fixed hydrogen concentration of 5 ppm was supplied for 15 min (T = 120 °C). The start of the gas supply corresponds to “0” on the time axis.

The results of a statistical study of the reproducibility and stability characteristics for gas sensitivity (mainly to hydrogen) of capacitive field-effect sensors in an initial TO-8 package were presented in [22], where, using the technology of pulsed laser deposition, it was shown that the yield of suitable sensors was not lower than 95%. Moreover, more than 90% of sensors have the following operating parameters at an operating temperature of T = 100 °C: sensitivity to a hydrogen concentration of 5 ppm is 1 ± 0.5 rel.u/ppm (noise level ± 0.05 rel.u., calculated value of detection threshold 150 ± 75 ppb) and the characteristic times of the response to the supply and removal of the concentration are τ_0.9_ = 5 ± 3 min and τ_0.1_ = 8 ± 5 min, respectively. Studies of the sensitivity to hydrogen for miniaturized sensors, by analogy with [22], were carried out on the experimental setups described in [23,24] using certified diluent generators and certified test gas mixtures. The number of experimental samples was 25 (the number of full-fledged ceramic packages after tomography and laser cutting). A summary of the resulting data is presented in Figure 9.

As can be seen in Figure 8, there was the following spread in the performance of fabricated capacitive sensors: the range of operating voltage measurements was (0.5 ± 0.4) V; the range of sensitivity to hydrogen was 0.4 ± 0.1 rel.u/m; the characteristic response times were τ_0.9_ = 5…7 min, and the characteristic relaxation times were τ_0.1_ = 7…15 min. Significant scatter explains the features of the occurrence of the main factors: (a) the error of measurable concentration generation (at least ± 10%) and (b) the technological factor that occurs with the inevitable scatter of the parameters and morphology of the deposited palladium films relative to the ablation product jet during pulsed laser deposition. Thus, the obtained result was in good agreement with the values previously reported in [22] and, like the invasive and non-invasive diagnostic methods presented above, verified the operability of the technology proposed in this article for the small-scale production of semiconductor field-effect gas sensors.

On the basis of the experimental data, it can be confirmed that the set tasks were completed. The 3D design and fabrication of heater and package products occurs simultaneously, which makes it possible to extremely quickly manufacture unique solutions for various types of field sensors without looking back at standard solutions (primarily the type of package) and according to unique researcher requirements (the number of microheaters, the size of hot spots, the number of contacts, the hole diameters in the cap of the package, etc.). The low cost of equipment for PLD deposition and laser micromilling, the absence of strict requirements for clean rooms, and the resistance of the ceramics to high temperatures and harsh operating conditions make the technology proposed in our work attractive for use in field-effect sensor applications.

## 5. Conclusions

It is worth noting that the field hydrogen sensor described in this paper is still being improved and is not yet optimal in terms of its functionality, in particular due to the still relatively large overall dimensions (7 × 6 × 2 mm, 0.1 cm^3^, compared with the original ø12 × 7 mm, 0.8 cm^3^), the need for heating and thermal stabilization of the Si-based structure, and, consequently, the relatively high power consumption (at least 0.4 W at 150 °C). Therefore, to solve the problems in detecting high concentrations of hydrogen (1000 ppm and higher), sensors that operate at ambient temperature or close to it [25,26] and do not require vacuum technologies for manufacturing are currently more promising. The response times of such structures to the concentration of hydrogen is a few to tens of seconds, which is an advantage, but a potential disadvantage is the peculiarities of working with the ambient temperature in real conditions, which is always changing and can often be very sharp (wind blowing, snowing, and raining). Therefore, in this work, in addition to the mechanical protection of the gas-sensitive structure, an emphasis was also placed on the very topical issue of thermal stabilization around one temperature point (even if it is several degrees higher than the surrounding one, which will probably be useful for any type of application of field-effect sensors). This temperature stabilization eliminates response uncertainty and drift (especially those associated with highly sensitive field-effect structures), making the sensor more convenient and predictable to use. Thus, the technology described in this article currently occupies an intermediate position between laboratory samples tested in clean rooms with a stable ambient atmosphere, and experimental and small-scale production sensors designed for real operating conditions to solve the narrow application of measuring low concentrations of hydrogen.

By combining various processing and deposition methods of materials (laser micromilling, inkjet printing, and sputtering), it was possible to manufacture the SMD metal–ceramic package for a fully functional solid-state field-effect semiconductor hydrogen sensor. The manufacture of the metal–ceramic package was based on a full digital 3D design. The great advantage of the combination of the described methods of material processing was the avoidance of working with high-temperature sintering processes of materials, generating a fundamental contribution to the destruction of the original geometric dimensions of the structure due to the shrinkage of the material. The characterization methods used in this work (industrial tomography, SEM microscopy, and thermal imaging) are relatively expensive instrumental methods against non-contact laser and optical profilometry as well as “destructive laser cutting” but confirm the accuracy of the result in comparison with a 3D model of a metal–ceramic package. In fact, there is no need to always apply the above-described expensive characterization methods since by using invasive and non-invasive digital control methods in parallel you can verify their convergence and then use only destructive and optical control, in an effort to save the main non-renewable resource: the time needed for the development and production of metal–ceramic packages. A statistical analysis of the results of a study of the gas sensitivity of miniaturized capacitive field-effect sensors also confirmed the operability of the proposed laser-micromilling-based technology for the small-scale production of field-effect gas sensors that combine both sensitive and actuating structural elements.

## Data Availability

Data sharing not applicable.
